# Different Effects of Three Polymorphisms in MicroRNAs on Cancer Risk in Asian Population: Evidence from Published Literatures

**DOI:** 10.1371/journal.pone.0065123

**Published:** 2013-06-04

**Authors:** Yeqiong Xu, Ling Gu, Yuqin Pan, Rui Li, Tianyi Gao, Guoqi Song, Zhenlin Nie, Liping Chen, Shukui Wang, Bangshun He

**Affiliations:** 1 The Central Laboratory of Nanjing First Hospital, Nanjing Medical University, Nanjing, China; 2 Department of Life Sciences, Nanjing Normal University, Nanjing, Jiangsu Province, China; Cleveland Clinic Lerner Research Institute, United States of America

## Abstract

MicroRNAs (miRNAs) are a class of small non-protein-coding RNAs, which have emerged as integrated and important post-transcriptional regulators of gene expression. It has been demonstrated that single nucleotide polymorphisms (SNPs) exist in protein-coding genes. Accumulated studies have evaluated the association of miRNA SNPs with cancer risk, especially in Asian population, which included a series of related studies. However, the results remain controversial for the different genetic backgrounds, living habits and environment exposed. To evaluate the relationship between SNPs in miRNAs and cancer risk, 21 studies focused on Asian population were enrolled for the pooled analysis for three polymorphisms rs2910164, rs11614913, rs3746444 in three miRNAs miR-146aG>C, miR-196a2C>T, miR-499A>G using odds ratios (ORs) with 95% confidence intervals (CIs). For rs2910164 polymorphism, C allele was observed association with decreased overall cancer risk. In addition, subgroup analysis revealed of rs2910164 C allele decreased hepatocellular carcinoma (HCC), cervical cancer and prostate cancer risk among Chinese population. For rs11614913 polymorphism, TT genotype was observed to be associated with decreased cancer risk, especially for cancer type of colorectal cancer (CRC), lung cancer and country of Korea, North India. Whereas, rs3746444 G allele was an increased cancer risk factor in Chinese population, especially for breast cancer. In conclusion, this meta-analysis indicated that rs2910164 C allele was associated with decreased cancer risk in Chinese population. However, the association varied from different cancer types. Furthermore, TT genotype of rs11614913 was associated with decreased cancer risk. While different cancer types and countries contributed to different effects. Whereas, rs3746444 G allele was a risk factor in Chinese population, and the association varied from different cancer types.

## Introduction

MicroRNAs (MiRNAs) are a family of naturally occurring, small noncoding RNAs of 21–24 nucleotides in length that regulate gene expression by base pairing with target mRNAs at the 3′UTR, leading to mRNA cleavage or translational repression [1], [2]. MiRNAs encoded in the genome are transcribed by RNA polymerase II or RNA polymerase III in the nucleus, where they cleaved by Drosha and Dicer sequentially [3], [4]. It has been suggested that miRNAs are important post-transcriptional regulators of gene expression that control diverse physiological and pathological processes. Accumulating evidence indicates that aberrant expressions of miRNAs were indicated to involve in tumorigenesis, development and prognosis of many cancers [5], [6], [7], [8], [9].

Single nucleotide polymorphisms (SNPs) were found in most genes, and recently SNPs of miRNAs have been paid much more attention. Studies have reported that miRNA SNPs could alter expressions or functions of miRNAs, and related to cancer risk. Meanwhile, studies have reported polymorphisms in miRNA genes, biogenesis pathway of miRNAs and their target binding sites. Moreover, polymorphisms in miRNA genes could directly influence the expressions and functions of miRNAs. Recently, miR-146aG>C (rs2910164), miR-196a2C>T (rs11614913) and miR-499A>G (rs3746444) were drawed close attention and were expected to demonstrate the association with many cancers [10], [11], [12], [13], [14], [15], [16], [17]. However, the results were generally inconsistent and inconclusive. Therefore, this meta-analysis focused on these three polymorphisms to deliberate their associations with cancer risk, which have surveyed in many populations. According to the recent studies, consistent conclusions were observed in Caucasian population while conflicting results were found in Asian population [13], [18], [19], [20], [21], [22], [23] due to the different countries, the numbers of study population, cancer types. To draw a conclusion of three polymorphisms and cancer risk in Asian population, an analysis of pooled published studies was required. This meta-analysis explored the associations between polymorphisms of three miRNAs and cancer in Asian population.

## Materials and Methods

### Literature and Inclusion Criteria

Using the combined words “miR-146a/miR-196a2/miR-499”, “cancer” or “carcinoma”, “genetic variation” or “polymorphism”, a comprehensive systematic bibliographic searching was applied through the medical databases PubMed, EMBASE and Web of Science for all medical published up to October 15th, 2012. In addition, studies were identified by manual search of the reference listed in the retrieved studies. Data from studies were accepted in our meta-analysis only if the study met all of the following criteria: (1) published in English; (2) available cancer risk and miR-146a/miR-499/miR-196a2 polymorphism data related to Asian population; (3) case-control studies; (4) sources of cases and sufficient available data to estimate an odds ratio (OR) with 95% confidence interval (CI); (5) available genotype frequency. Moreover, the studies were eliminated if there are no raw data in the studies, or they are case-only studies, case reports, editorials, and review articles (including meta-analyses).

### Data Extraction

Information was reviewed carefully extracted from all the eligible articles independently by two of the authors (Yeqiong Xu and Ling Gu) according to the inclusion criteria listed above. The characteristics information of enrolled studies was extracted from the study: the first author’s last name, year of publications, country of subjects, cancer type, the source of controls, genotyping method, matching numbers of genotyped cases and controls, polymorphism site and *P* for HWE (Table 1). If discrepancies and differences were existed after data collection, discussion was carried out to get consensus.

**Table 1:** Characteristics of studies included in the meta-analysis.

Author	Year	Country	Cancer type	Control Source	Method	Patient	Health	Case/Control	Polymorphism site	*P* for HWE
Xu	2008	China	HCC	PB	PCR-RFLP	479 HCC patients were diagnosed histopathologically, lived in Guangzhou or the surrounding regions, mean age (SD) 45.2(12.1)	504 cancer-free controls were collected in the same period as patients, frequency-matched to the cases on age and sex, mean age (SD) 44.6(11.4)	479/504	rs29010164	0.119
Hu	2008	China	Breast cancer	PB	PCR-RFLP	1009 newly diagnosed and histopathologically confirmed breast cancer patients from Nanjing, including 998 invasive, 28 ductal carcinoma, and 3 lobular carcinoma, mean age (SD) 51.60(11.08)	1093 cancer-free control women, frequency-matched to the cases on age and residential area, mean age (SD) 51.77(11.19)	1009/1093	rs11614913,rs29010164,rs3746444	0.207,0.221,0.057
Tian	2009	China	Lung cancer	PB	PCR-RFLP	1058 lung cancer patients were histopathologically diagnosed, lived in Nanjing, without the restrictions of age, sex, and histology, mean age (SD) 59.78(10.04)	1035 cancer-free controls conducted in Jiangsu Province during the same period as the cases were recruited. The control subjects had no history of cancer and were frequency matched to the cases on age, sex, and residential area, mean age (SD) 59.66(9.83)	1058/1035	rs11614913,rs29010164,rs3746444	0.700,0.853,0.404
Guo	2010	China	Esophageal cancer	PB	SNPshot	444 ESCC patients were from Chongqing City and the surrounding regions and were histopathologically diagnosed without the restrictions of age and sex	468 Cancer-free controls, having no history or family history of cancer and other genetic disease, and were frequency matched to the cases on age, gender, and residential area	444/468	rs29010164	0.12
Zeng	2010	China	Gastric cancer	HB	PCR-RFLP	304 gastric cancer patients (mean age 59, age range 51–66) were from Jiangsu Province, and all confirmed by endoscopic biopsy or surgical specimens	304 cancer-free controls (mean age 58, age range 50–66) matched to gastric cancer cases by gender and age, were selected from patients hospitalized	304/304	rs29010164	0.122
Srivastava	2010	North Indian	Gallbladder cancer	PB	PCR-RFLP	230 gallbladder cancer patients were diagnosis and confirmed for all cases by fine needle aspirated cell cytology and histopathology	230 control subjects were healthy adults without a history of cancer, who were randomly selected from general population and were frequency matched to cancer cases on age and gender	230/230	rs11614913,rs29010164,rs3746444	0.068,0.080,0.566
Xu	2010	China	Prostate cancer	PB	PCR-RFLP	251 prostate cancer patients were confirmed by biopsy, lived in Nanjing	280 controls were age-matched, and the subjects were healthy checkup examinees without cancer history and were collected in the same period	251/280	rs29010164	0.191
Chen	2010	China	CRC	PB	PCR–LDR	126 CRC patients had undergone surgery and been histopat hologically confirmed.The mean age was 57.9.All cases were ethnically Chinese	407 controls were free of disease on health check-up. They were matched with the case patients by age and sex. The mean age was 55.6. All controls were ethnically Chinese	126/407	rs11614913	0.789
Li	2010	China	HCC	HB	PCR-RFLP	310 cirrhosis patients (mean age 49) with HCC served as cases were diagnosed via histopathology. The subjects were all Han Chinese	222 cirrhosis patients (mean age 50) without HCC served as controls. The subjects were all Han Chinese	310/222	rs11614913	0.402
Yoo	2010	Korea	Lung cancer	PB	Melting curve analysis	654 newly diagnosed lung cancer patients included 287 squamous cell carcinomas, 246 adenocarcinomas, 10 large cell carcinomas, and 101 small cell carcinomas. There were no gender, histologic, or stage restrictions, mean age (SD) 61.1(9.0). All patients were ethnic Koreans who resided in Daegu City or the surrounding regions	640 control subjects were frequency-matched to the cases based on gender and age, mean age (SD) 60.5(9.4). All controls were ethnic Koreans who resided in Daegu City or the surrounding regions	654/640	rs11614913	0.126
Dou	2010	China	Glioma	PB	PCR–LDR	670 newly diagnosed glioma cancer patients confirmed via histopathology, including 246 astrocytomas, 204 glioblastoma, 193 other gliomas. All the subjects were Han Chinese origin. Among them, 643 cases were genotyped successfully	680 cancer-free controls were frequency matched to the cases with the same age, sex, and residence area. All the subjects were Han Chinese origin.Among them, 656 controls were genotyped successfully	643/656	rs11614913	0.119
Qi	2010	China	HCC	PB	PCR–LDR	361 HCC patients (mean age 49) with chronic HBV infection were designated as cases. The diagnosis of HCC was histopathologically confirmed, All subjects were Han Chinese	391 healthy volunteers (mean age 35) served as healthy controls. All subjects were Han Chinese	361/391	rs11614913	0.869
Peng	2010	China	Gastric cancer	PB	PCR-RFLP	213 gastric cancer were inpatients newly diagnosed and histopathologically confirmed. The subjects in this study were unrelated Han Chinese, mean age (SD) 58(12)	213 cancer-free controls had no current or previous diagnosis of cancer and were frequency matched to cases on age and gender. The subjects in this study were unrelated Han Chinese, mean age (SD) 58.3(11.8)	213/213	rs11614913	0.936
Zhou	2011	China	HCC	PB	PCR-RFLP	186 patients with primary liver cancer were diagnosed either by histopathologic or imaging evidence, mean age (SD) 52.10(15.20)	483 healthy individuals undergoing routine medical examination without any medical illness, matched with patients by age and gender	186/483	rs29010164,rs3746444	0.056,0.100
Yue	2011	China	Cervical cancer	PB	PCR-RFLP	447 cervical cancer patients were newly diagnosed and histologically confirmed. All subjects were genetically-unrelated Han Chinese, mean age (SD) 46.38(8.98)	443 cancer-free controls consisted of women in good health and with no malignancy history. They were frequency-matched to the cases by age, with people who were being recruited during the same time. All subjects were genetically-unrelated Han Chinese, mean age (SD) 46.38(8.98)	447/443	rs29010164	0.285
Mittal	2011	North India	Bladder cancer	PB	PCR-RFLP	212 histologically confirmed patients with UBC (mean age 59.0 years; 187 men and 25 women) were unrelated North Indian.	250 healthy and genetically unrelated were recruited as the control (mean age 57.8 years, 215 men and 35 women). All the controls were age, sex matched, of similar ethnicity, and had no evidence of malignancy or chronic disease	212/250	rs11614913,rs29010164,rs3746444	**0.003,0.007,0.020**
Zhou	2011	China	Cervical cancer	PB	PCR-RFLP	226 unrelated female patients ranging in age from 23 to 75, mean (SD) 44.96 (9.48). The diagnosis of CSCC was confirmed in all cases by histological examination of tissue from biopsy or resected specimens. All subjects were Han population living in Sichuan province of southwest China	309 healthy women was selected randomly from a routine health survey in the same hospital according to the age distribution of individuals with CSCC	226/309	rs11614913,rs29010164,rs3746444	0.077,0.060,**0.005**
Okubo	2011	Japan	Gastric cancer	HB	PCR-RFLP	552 gastric cancer patients was diagnosed histologically and was classified according to Lauren’s classification, mean(SD ) 64.4(11.2)	697 non-cancer subjects had no evidence of GC by upper gastroscopy, 214 subjects were diagnosed as having ulcer diseases including 141 GU and 73 DU, while 483 subjects were diagnosed as non-ulcer subjects, mean(SD ) 61.0(13.5)	552/697	rs11614913,rs29010164,rs3746444	0.510,0.278,**0.048**
Hishida	2011	Japan	Gastric cancer	HB	PCR-CTPP	583 of the cases diagnosed as gastric cancer, mean(SD) 58.8(10.5)	1637 cancer-free outpatients (controls) were age- and sex-frequency matched with cases, mean(SD) 58.7(10.6)	583/1637	rs29010164	0.738
George	2011	North Indian	Prostate cancer	PB	PCR-RFLP	159 prostate cancer patients were histologically confirmed, mean(SD) 66.6(6.22)	230 controls matched each case patient in age (65.8±7.29) from a population of healthy men	159/230	rs11614913,rs29010164,rs3746444	**0.002,0.002**,0.073
Min	2011	Korea	CRC	PB	PCR-RFLP	446 CRC patients included 147 proximal colon cancer, 104 distal colon cancer, 185 rectal cancer, 11 ixed colorectal cancer, mean age(SD) 61.89(12.35)	502 controls randomly selected following a heath screening which were age and gender matched with cases, mean age(SD) 61.74(12.11)	446/502	rs11614913,rs29010164,rs3746444	0.633,0.443,0.453
Zhu	2011	China	CRC	PB	Taqman	573 newly diagnosed CRC patients were histopathologically confirmed, mean age(SD) 60.3(12.5)	588 cancer-free controls were genetically unrelated to the cases without individual history of cancer, and frequency matched to patients based on sex and age, mean age(SD) 59.3(9.8)	573/588	rs11614913	0.79
Hong	2011	Korea	Lung cancer	HB	Taqman	406 lung cancer patients were histopathologically diagnosed as having NSCLC, mean age(SD) 67.3(10.2)	428 cancer-free controls were recruited from among the residents of Busan city, and frequency matched to patients based on sex and age, mean age(SD) 63.2(10.2)	406/428	rs11614913	0.163
Zhan	2011	China	CRC	HB	PCR-RFLP	252 CRC patients (mean age 54.8) were in-patien ts with newly diagnosed and histopathologically confirmed. All subjects were unrelated Han Chinese	543 cancer-free control subjects (mean age 53.2) had no current or previous diagnosis of cancer and were frequently age or gender matched to cases. All subjects were unrelated Han Chinese	252/543	rs11614913	0.849
Zhang	2011	China	Breast cancer	PB	PCR-RFLP	252 breast cancer women were recruited without any restrictions on age, sex or disease histology, and were collected in Jiashan County, mean age(SD) 54.66(11.18)	248 controls were enrolled from the cancer-free population matched with cases by age, sex and residence area, mean age(SD) 54.51(11.41)	252/248	rs11614913	0.893
Xiang	2012	China	HCC	PB	PCR-RFLP	100 HCC patients without any other types of liver diseases histopathologically confirmed, including 27 without HBV and 73 with HBV, mean age(SD) 48.55(9.29)	100 healthy controls were matched with age, mean age(SD) 45.12(15.82)	100/100	rs29010164,rs3746444	0.506,0.284
Kim	2012	Korea	HCC	PB	PCR-RFLP	159 HCC patients were included. The clinical stage of HCC was evaluated on the basis of the TNM classification and OKUDA stage system, mean age(SD) 56.06(11.02)	201 controls selected from health screening program participants to exclude those with a history of cancer and other medical diseases, were matched with age and sex, mean age(SD) 53.58(11.17)	159/201	rs11614913,rs29010164,rs3746444	0.356,0.190,0.278
Zhou	2012	China	Gastric cancer	HB	TaqMan	750 gastric patients from Nanjing and 936 patients from Yixing served as cases. All the patients were newly diagnosed with histopathologically confirmed. All subjects are ethnic Han Chinese	835 healthy from Nanjing and 1060 healthy from Yixing served as controls, were age- and sex- matched with cases. All subjects are ethnic Han Chinese	1686/1895	rs29010164	0.641
Lung	2012	China	Nasopharyngeal cancer	PB	Melting curve analysis	233 nasopharyngeal cancer patients were from HongKong, mean age(SD) 51.3(11.3)	173 sex- and age- matched healthy selected from HongKong. Participants in all control groups had no cancer history, mean age(SD) 49.5(10.0)	233/173	rs29010164	0.106
Alshatwi	2012	Saudi Arabia	Breast cancer	PB	TaqMan	100 breast cancer patients included 58 premenopausal (mean age 37.5) and postmenopausal (mean age 61.2)	100 healthy controls were matched with age	100/100	rs11614913,rs29010164,rs3746444	**0.032**,0.051,0.227
Chu	2012	China	Oral cancer	HB	PCR-RFLP	470 male patients from Taiwan were included.	425 male controls matched with age were enrolled from the physical examination in the hospitals as cases, had neither self reported history of cancer of any sites	470/425	rs11614913,rs29010164,rs3746444	0.686,0.939,0.975

HCC: hepatocellular carcinoma; CRC: colorectal cancer; HB: hospital based; PB: population based; PCR-RFLP: polymerase chain reaction–restriction fragment length polymorphism; PCR-CTPP: polymerase chain reaction with confronting two-pair primers; PCR-LDR: ligation detection reaction; HWE: Hardy-Weinberg equilibrium.

### Statistical Analysis

The strength of association between the three SNPs and cancer risk was assessed by odds ratios (ORs) with 95% confidence intervals (CIs). The pooled ORs were estimated for dominant model, recessive model, homozygote comparison, heterozygote comparison and allelic comparison, respectively. Stratified analyses were also performed by cancer type (HCC, CRC, cervical cancer, prostate cancer, breast cancer, gastric cancer, lung cancer, and other cancers group which combined the cancer types containing less than two individual studies), country (China, Korea, Japan, North India and other countries group which combined the countries containing less than two individual studies) source of control and genotyping method. Heterogeneity across the studies was evaluated by using the Chi-square test based Q-statistic test, and it was considered significant when *P_heterogeneity_* (*P*_h_) <0.05. The data were combined using both fixed-effects (the Mantel-Haenszel method) and random effects (the DerSimonian and Laird method) models. A random-effect model was employed when heterogeneity existed [24]–[25], Otherwise, the fixed-effect model was employed to pool the results [26]. Moreover, to assess the stability of the results, a sensitivity analysis was performed. Publication bias was checked graphically by using funnel plots and statistically using the Egger's linear regression test. For the controls of each study, the genotype frequencies of the three polymorphisms of miRNA were assessed for Hardy-Weinberg equilibrium using a web-based program (http://ihg.gsf.de/cgi-bin/hw/hwa1.pl). All statistical tests were performed with STATA 10.0 and all the *P* values were two-sided.

## Results

### Characteristics of Studies

**Figure 1:**
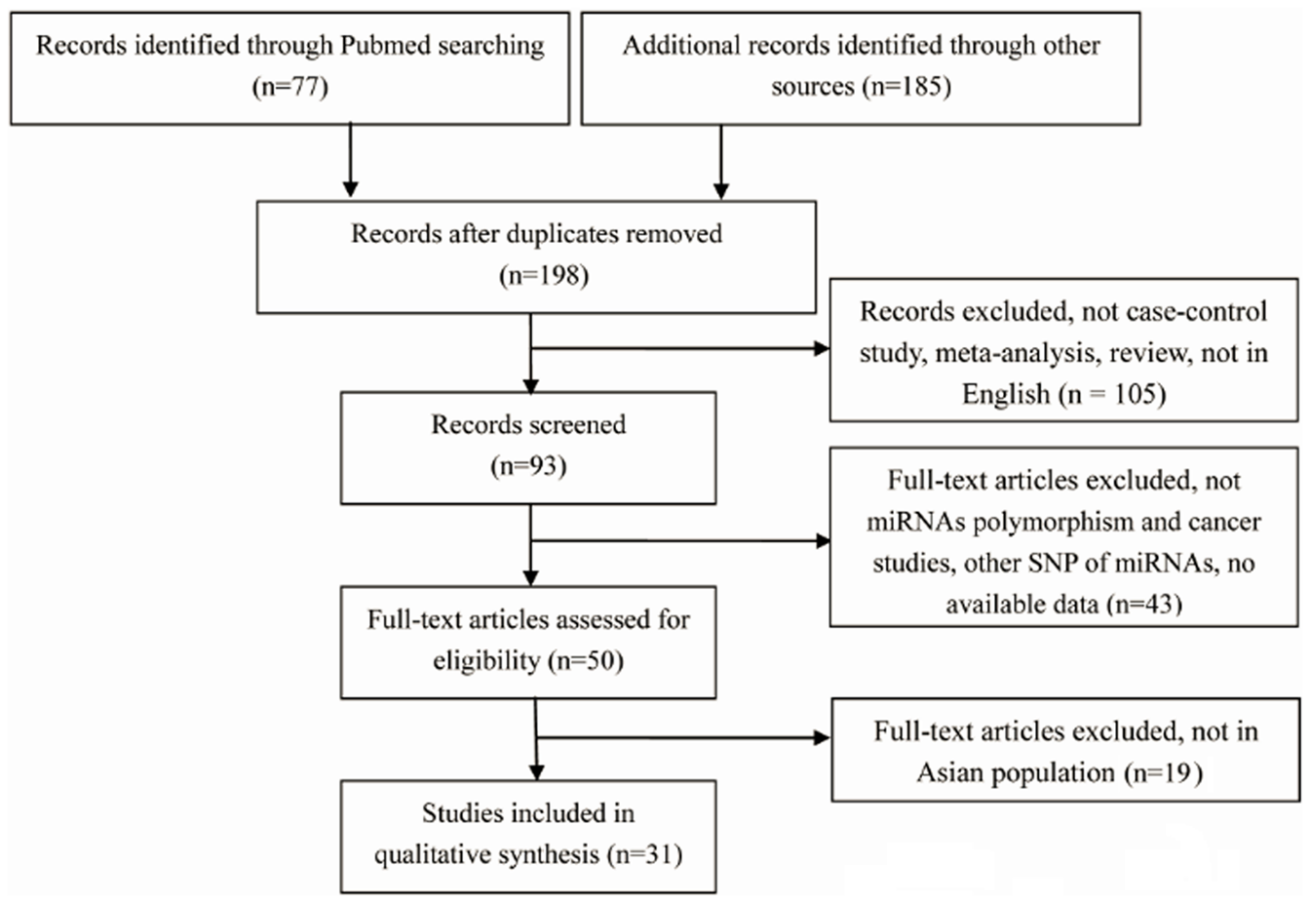
Flow chart of studies identified according to inclusion and exclusion criteria.

This study enrolled 31 eligible papers (Figure 1) according to the inclusion criteria. For rs2910164 polymorphism, 21 studies with available data were enrolled in the pooled analysis. These studies consisted of China (13 studies), Korea (2 studies), North India (3 studies) and Japan (2 studies) related to HCC (4 studies), gastric cancer (4 studies), cervical cancer (2 studies), prostate cancer (2 studies), breast cancer (2 studies) and other cancers (7 studies). In addition, the controls of most studies were population-based, and the main genotyping method was PCR-RFLP (Table 1).

For rs11614913 polymorphism, 21 studies provided available data, which were classified into CRC (4 studies), HCC (3 studies), breast cancer (3 studies), lung cancer (3 studies), gastric cancer (2 studies) and other cancers (6 studies). Meanwhile, these studies with data of 12 studies of Chinese population, 4 studies of Korean population, 3 studies of North Indian population and 2 studies of other countries. To analyze polymorphisms, genotyping by polymerase chain reaction-restriction fragment length polymorphism (PCR-RFLP) were performed by the most studies, in which 16 were population-based and 5 were hospital-based (Table 1).

For rs3746444 polymorphism, 13 studies covered China (6 studies), Korea (2 studies), North India (3 studies) and other countries (2 studies) related to HCC (3 studies), breast cancer (2 studies) and other cancers (8 studies) were included in the pooled analysis. What's more, these studies contained 11 of population-based controls and 2 of hospital-based controls.

### Main Results

**Figure 2:**
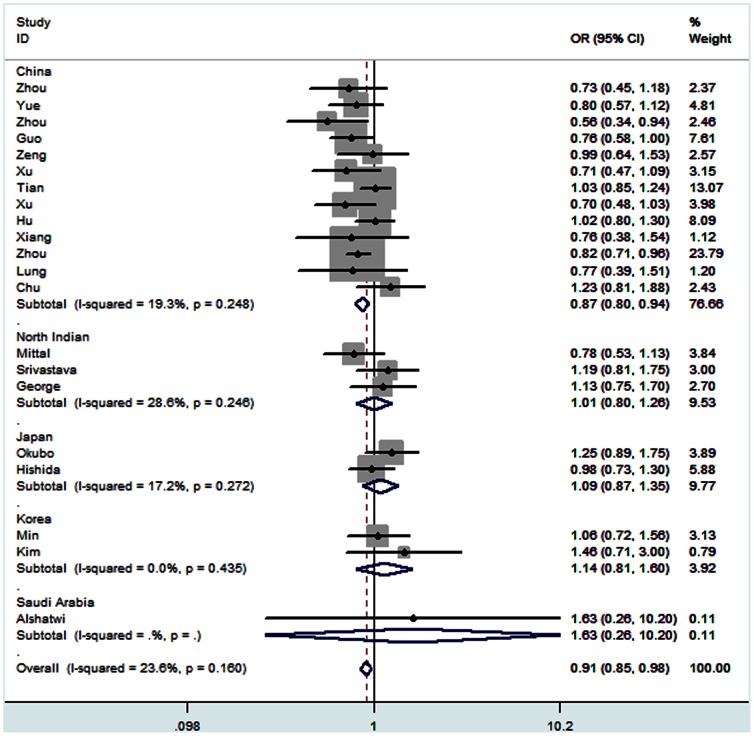
Forest plots of effect estimates for rs2910164 stratified by country (GC vs GG). For each studies, the estimate of OR and its 95% CI is plotted with a *box* and a *horizontal line*. *Filled diamond* pooled OR and its 95% CI.

For rs2910164 polymorphism, results of pooled analysis revealed significantly decreased risk was observed for the the comparison of homozygote model (CC vs GG: OR = 0.83, 95% CI: 0.70–0.99, *P*_h_ = 0.000), heterozygote model (GC vs GG: OR = 0.91, 95% CI: 0.85–0.98, *P*_h_ = 0.160) (Figure 2) and dominant model (CC+GC vs GG: OR = 0.89, 95% CI: 0.80–1.00, *Z* = 2.06, *P* = 0.040, *P*_h_ = 0.004). Cancer subgroup analysis revealed an obvious decreased risk was found in cervical cancer for all four comparison models (CC vs GG: OR = 0.50, 95% CI: 0.37–0.68, *P*_h_ = 0.814; GC vs GG: OR = 0.72, 95% CI: 0.55–0.95, *P*_h_ = 0.254; CC+GC vs GG: OR = 0.63, 95% CI: 0.49–0.82, *P*_h_ = 0.382; CC vs GG+GC: OR = 0.65, 95% CI: 0.52–0.82, *P*_h_ = 0.359). Similarly, decreased cancer risk was observed when compared of homozygote model and recessive model in prostate cancer (CC vs GG: OR = 0.54, 95% CI: 0.34–0.87, *P*_h_ = 0.425; CC vs GG+GC: OR = 0.65, 95% CI: 0.44–0.96, *P*_h_ = 0.699). Moreover, a decreased risk was observed in HCC for the comparison of homozygote model and dominant model (CC vs GG: OR = 0.75, 95% CI: 0.57–0.98, *P*_h_ = 0.213; CC+GC vs GG: OR = 0.77, 95% CI: 0.61–0.98, *P*_h_ = 0.284) as well. Country subgroup analysis revealed that rs2910164 C allele was associated with a decreased risk of cancer in Chinese population (CC vs GG: OR = 0.73, 95% CI: 0.60–0.88, *P*_h_ = 0.000; GC vs GG: OR = 0.87, 95% CI: 0.80–0.94, *P*_h_ = 0.248; CC+GC vs GG: OR = 0.81, 95% CI: 0.72–0.92, *P*_h_ = 0.032; CC vs GG+GC: OR = 0.83, 95% CI: 0.71–0.97, *P*_h_ = 0.000). Moreover, a significantly decreased risk was found for the comparison of homozygote model (CC vs GG: OR = 0.79, 95% CI: 0.64–0.98, *P*_h_ = 0.001), heterozygote model (GC vs GG: OR = 0.90, 95% CI: 0.83–0.99, *P*_h_ = 0.232) and dominant model (CC+GC vs GG: OR = 0.86, 95% CI: 0.75–0.98, *P*_h_ = 0.021) in population-based controls. Finally, genotyping method subgroup analysis revealed a decreased cancer risk determined by Taqman in all four comparion models (CC vs GG: OR = 0.70, 95% CI: 0.58–0.85, *P*_h_ = 0.450; GC vs GG: OR = 0.92, 95% CI: 0.85–0.98, *P*_h_ = 0.467; CC+GC vs GG: OR = 0.79, 95% CI: 0.69–0.91, *P*_h_ = 0.479; CC vs GG+GC: OR = 0.79, 95% CI: 0.67–0.93, *P*_h_ = 0.667), as summarized in Table 2.

**Table 2:** Stratified analyses of the miR-146aG>C (rs2910164) polymorphism and cancer risk.

Variables	n^a^	CC vs GG			GC vs GG					CC+GC vs GG						CC vs GG+GC						C allele vs G allele						
		OR(95%CI)	*P* b	*I^2^*	OR(95%CI)	*P* b		*I^2^*		OR(95%CI)		*P* b		*I^2^*		OR(95%CI)		*P* b		*I^2^*		OR(95%CI)		*P* b		*I^2^*		
Total	21	**0.83(0.70,0.99)** c	0.000	65.8	**0.91(0.85,0.98)**	0.160		23.6		**0.89(0.80,1.00)** c		0.004		50.7		0.89(0.79,1.01)^c^		0.000		65.4		**0.92(0.85,1.00)** c		0.000		69.3		
Cancer type																												
HCC	4	**0.75(0.57,0.98)**	0.213	33.1	0.79(0.61,1.02)		0.343		10.0		**0.77(0.61,0.98)**		0.284		21.0		0.88(0.73,1.05)		0.234		29.7		**0.88(0.78,1.00)**		0.245		27.8	
Cervical cancer	2	**0.50(0.37,0.68)**	0.814	0.0	**0.72(0.55,0.95)**		0.254		23.1		**0.63(0.49,0.82)**		0.382		0.0		**0.65(0.52,0.82)**		0.359		0.0		**0.72(0.62,0.84)**		0.796		0.0	
Prostate cancer	2	**0.54(0.34,0.87)**	0.425	0.0	0.91(0.67,1.22)		0.131		56.1		0.85(0.64,1.13)		0.062		71.4		**0.65(0.44,0.96)**		0.699		0.0		0.83(0.69,1.01)		0.071		69.3	
Breast cancer	2	1.00(0.77,1.29)	0.708	0.0	1.03(0.81,1.31)		0.619		0.0		1.02(0.81,1.28)		0.662		0.0		0.97(0.81,1.15)		0.745		0.0		0.99(0.88,1.11)		0.854		0.0	
Gastric cancer	4	0.92(0.63,1.34)^c^	0.000	84.1	0.91(0.81,1.02)		0.136		45.8		0.96(0.74,1.24)^c^		0.011		73.1		0.92(0.70,1.21)^c^		0.000		83.5		0.95(0.78,1.16)^c^		0.000		86.4	
Other cancers	7	0.97(0.72,1.32)^c^	0.030	57.0	0.96(0.85,1.09)		0.256		22.7		0.96(0.86,1.08)		0.119		40.8		1.00(0.75,1.34)^c^		0.002		71.6		0.99(0.85,1.14)^c^		0.004		69.1	
Country																												
China	13	**0.73(0.60,0.88)** c	0.000	66.7	**0.87(0.80,0.94)**		0.248		19.3		**0.81(0.72,0.92)** c		0.032		46.6		**0.83(0.71,0.97)** c		0.000		70.5		**0.87(0.79,0.95)** c		0.000		70.3	
Korea	2	0.98(0.69,1.39)	0.367	0.0	1.14(0.81,1.60)		0.435		0.0		1.07(0.77,1.48)		0.380		0.0		0.88(0.70,1.11)		0.658		0.0		0.96(0.82,1.12)		0.491		0.0	
North India	3	1.30(0.69,2.48)	0.382	0.0	1.01(0.80,1.26)		0.246		28.6		1.03(0.82,1.28)		0.195		38.7		1.29(0.68,2.44)		0.441		0.0		1.04(0.87,1.25)		0.204		37.1	
Japan	2	1.21(0.97,1.52)	0.069	69.8	1.09(0.87,1.35)		0.272		17.2		1.15(0.93,1.41)		0.123		57.9		1.14(0.98,1.32)		0.130		56.4		1.11(1.00,1.23)		0.058		72.3	
Source of controls																												
Population based	16	**0.79(0.64,0.98)** c	0.001	61.4	**0.90(0.83,0.99)**		0.232		19.4		**0.86(0.75,0.98)** c		0.021		46.6		0.88(0.76,1.03)^c^		0.001		61.5		0.91(0.83,1.00)^c^		0.000		65.1	
Hospital based	5	0.93(0.68,1.27)^c^	0.001	79.3	0.93(0.83,1.04)		0.114		46.4		0.98(0.78,1.23)^c^		0.014		67.8		0.91(0.73,1.13)^c^		0.001		78.5		0.95(0.81,1.12)^c^		0.000		81.9	
Genotyping method																												
PCR-RFLP	16	0.84(0.69,1.03)^c^	0.000	65.6	0.96(0.88,1.05)		0.153		26.9		0.91(0.80,1.04)^c^		0.007		52.9		0.88(0.77,1.00)^c^		0.002		58.7		0.92(0.84,1.01)^c^		0.000		66.7	
Taqman	2	**0.70(0.58,0.85)**	0.450	0.0	**0.92(0.85,0.98)**		0.467		0.0		**0.79(0.69,0.91)**		0.479		0.0		**0.79(0.67,0.93)**		0.667		0.0		**0.84(0.76,0.92)**		0.570		0.0	
Other methods	3	0.85(0.45,1.62)^c^	0.008	79.5	0.85(0.70,1.02)		0.441		0.0		0.85(0.71,1.01)		0.160		45.5		1.00(0.55,1.80)^c^		0.001		86.7		1.00(0.73,1.37)^c^		0.001		86.0	

HCC: hepatocellular carcinoma; PCR-RFLP: polymerase chain reaction–restriction fragment length polymorphism.

a Number of included studies.

b *P* value of *Q* test for heterogeneity test.

c Random-effect model was used when P value for heterogeneity <0.05; otherwise, fixed-effect model was used.

Statistically significant results were in bold.

**Figure 3:**
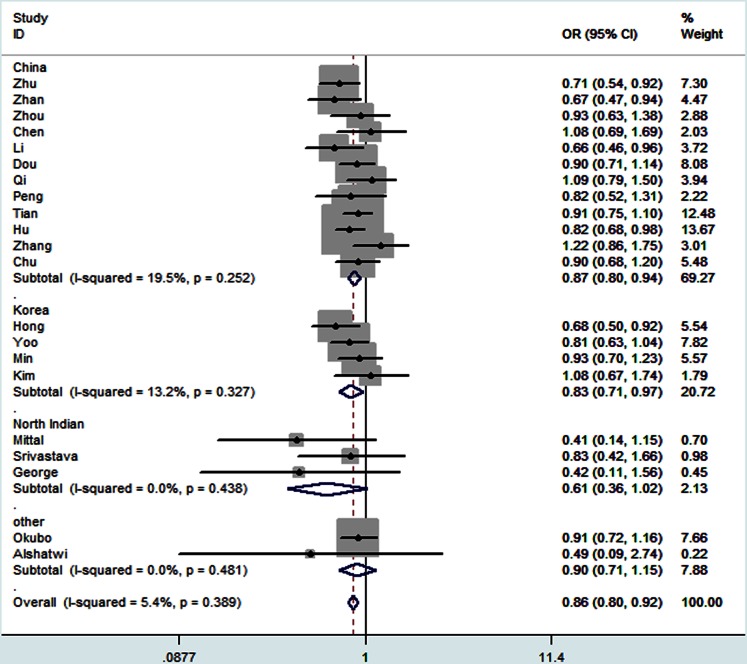
Forest plots of effect estimates for rs11614913 stratified by country (TT vs CC+CT). For each studies, the estimate of OR and its 95% CI is plotted with a *box* and a *horizontal line*. *Filled diamond* pooled OR and its 95% CI.

For rs11614913 polymorphism, decreased risk associations were observed in the overall pooled analysis for the comparison of homozygote model (TT vs CC: OR = 0.84, 95% CI: 0.74–0.95, *P*_h_ = 0.029) and recessive model (TT vs CC+CT: OR = 0.86, 95% CI: 0.80–0.92, *P*_h_ = 0.389) (Figure 3). Cancer types subgroup analysis revealed a significant association in the comparison of homozygote model (TT vs CC: OR = 0.70, 95% CI: 0.57–0.85, *P*_h_ = 0.284), heterozygote model (CT vs CC: OR = 0.81, 95% CI: 0.68–0.97, *P*_h_ = 0.367), dominant model (TT+CT vs CC: OR = 0.77, 95% CI: 0.65–0.91, *P*_h_ = 0.377) and recessive model (TT vs CC+CT: OR = 0.80, 95% CI: 0.69–0.94, *P*_h_ = 0.198) in colorectal cancer. Similarly, a decreased risk was observed for the comparison of homozygote model (TT vs CC: OR = 0.77, 95% CI: 0.65–0.91, *P*_h_ = 0.895), dominant model (TT+CT vs CC: OR = 0.85, 95% CI: 0.74–0.98, *P*_h_ = 0.289) and recessive model (TT vs CC+CT: OR = 0.83, 95% CI: 0.73–0.95, *P*_h_ = 0.281) in lung cancer and homozygote model (TT vs CC: OR = 0.79, 95% CI: 0.63–0.99, *P*_h_ = 0.127) in breast cancer. In contrast, an increased risk was observed in other cancers (CT vs CC: OR = 1.49, 95% CI: 1.28–1.74, *P*_h_ = 0.178; TT+CT vs CC: OR = 1.39, 95% CI: 1.20–1.61, *P*_h_ = 0.226). Subgroup analysis by country revealed a decreased risk for the comparison of recessive model in China (TT vs CC+CT: OR = 0.87, 95% CI: 0.80–0.94, *P*_h_ = 0.252) and Korea (OR = 0.83, 95% CI: 0.72–0.97, *P*_h_ = 0.327). In addition, the decreased risk was also observed for comparison of homozygote model (TT vs CC: OR = 0.77, 95% CI: 0.64–0.93, *P*_h_ = 0.616) and dominant model (TT+CT vs CC: OR = 0.84, 95% CI: 0.72–0.98, *P*_h_ = 0.162) in Korea. However, an increased risk was observed in North India (CT vs CC: OR = 1.53, 95% CI: 1.22–1.93, *P*_h_ = 0.832; TT +CT vs CC: OR = 1.43, 95% CI: 1.15–1.79, *P*_h_ = 0.796). Subgroup analysis by the source of control revealed significant decrease risk for the comparison of recessive model not only in the hospital-population based controls (TT vs CC+CT: OR = 0.79, 95% CI: 0.69–0.90, *P*_h_ = 0.295) but also in population-based controls (TT vs CC+CT: OR = 0.88, 95% CI: 0.81–0.95, *P*_h_ = 0.509), and a decreased risk for the comparison of homozygote model (TT vs CC: OR = 0.82, 95% CI: 0.74–0.91, *P*_h_ = 0.226) was revealed in population-based controls as well. Subgroup analysis determined by genotyping method showed a significant association between the polymorphism and cancer risk in both PCR-RFLP and Taqman group for the comparison of homozygote model (TT vs CC: OR = 0.81, 95% CI: 0.69–0.96, *P*_h_ = 0.044; OR = 0.71, 95% CI: 0.55–0.91, *P*_h_ = 0.740, respectively) and recessive model (TT vs CC+CT: OR = 0.87, 95% CI: 0.80–0.94, *P*_h_ = 0.444; OR = 0.69, 95% CI: 0.57–0.85, *P*_h_ = 0.903, respectively), as summarized in Table 3.

**Table 3:** Stratified analyses of the miR-196a2C>T (rs11614913) polymorphism and cancer risk.

Variables	n^a^	TT vs CC			CT vs CC			TT+CT vs CC			TT vs CC+CT			T allele vs C allele			
		OR(95%CI)	*P* b	*I^2^*	OR(95%CI)	*P* b	*I^2^*	OR(95%CI)	*P* b	*I^2^*	OR(95%CI)	*P* b	*I^2^*	OR(95%CI)	*P* b	*I^2^*	
Total	21	**0.84(0.74,0.95)** c	0.029	40.4	1.05(0.92,1.20)^c^	0.000	66.5	1.00(0.88,1.14)^c^	0.000	66.9	**0.86(0.80,0.92)**	0.389	5.4	0.94(0.88,1.00)^c^	0.007	48.3	
Cancer type																	
CRC	4	**0.70(0.57,0.85)**	0.284	21.1	**0.81(0.68,0.97)**	0.367	5.2	**0.77(0.65,0.91)**	0.377	3.1	**0.80(0.69,0.94)**	0.198	35.7	**0.84(0.76,0.92)**	0.281	21.6	
HCC	3	0.89(0.67,1.17)	0.088	58.9	0.95(0.74,1.21)	0.643	0.0	0.92(0.73,1.17)	0.277	22.2	0.92(0.74,1.14)	0.113	54.1	0.94(0.82,1.08)	0.069	62.5	
Breast cancer	3	**0.79(0.63,0.99)**	0.127	51.5	0.96(0.78,1.17)	0.059	64.6	1.18(0.68,2.03)^c^	0.028	72.0	0.89(0.75,1.04)	0.114	53.9	1.05(0.78,1.40)^c^	0.028	72.1	
Gastric cancer	2	0.80(0.61,1.06)	0.306	4.5	0.84(0.65,1.08)	0.163	48.5	0.82(0.65,1.04)	0.162	48.8	0.89(0.72,1.11)	0.698	0.0	0.89(0.78,1.02)	0.230	30.5	
Lung cancer	3	**0.77(0.65,0.91)**	0.895	0.0	0.90(0.77,1.04)	0.098	57.0	**0.85(0.74,0.98)**	0.289	19.4	**0.83(0.73,0.95)**	0.281	21.3	**0.87(0.80,0.95)**	0.854	0.0	
Other cancers	6	1.12(0.91,1.38)	0.238	26.2	**1.49(1.28,1.74)**	0.178	34.4	**1.39(1.20,1.61)**	0.226	27.8	0.87(0.75,1.02)	0.624	0.0	1.08(0.99,1.19)	0.752	0.0	
Country																	
China	12	0.87(0.72,1.05)^c^	0.002	62.8	0.99(0.83,1.18)^c^	0.001	66.4	0.94(0.79,1.12)^c^	0.000	69.0	**0.87(0.80,0.94)**	0.252	19.5	0.92(0.85,1.00)^c^	0.007	57.5	
Korea	4	**0.77(0.64,0.93)**	0.616	0.0	0.89(0.75,1.05)	0.053	60.9	**0.84(0.72,0.98)**	0.162	41.6	**0.83(0.72,0.97)**	0.327	13.2	**0.87(0.79,0.96)**	0.608	0.0	
North India	3	0.74(0.44,1.26)	0.571	0.0	**1.53(1.22,1.93)**	0.832	0.0	**1.43(1.15,1.79)**	0.796	0.0	0.61(0.36,1.02)	0.438	0.0	1.17(0.99,1.38)	0.880	0.0	
Other countries	2	0.87(0.63,1.20)	0.749	0.0	1.07(0.82,1.40)	0.093	64.6	1.03(0.80,1.33)	0.096	63.8	0.90(0.71,1.15)	0.481	0.0	0.97(0.84,1.13)	0.236	28.9	
Source of controls																	
Population based	16	**0.82(0.74,0.91)**	0.226	20.0	1.03(0.89,1.20)^c^	0.000	63.8	1.00(0.86,1.15)^c^	0.000	64.0	**0.88(0.81,0.95)**	0.509	0.0	0.96(0.89,1.03)^c^	0.028	44.6	
Hospital based	5	0.82(0.58,1.16)^c^	0.005	72.9	1.10(0.78,1.53)^c^	0.002	76.3	0.99(0.71,1.37)^c^	0.001	78.2	**0.79(0.69,0.90)**	0.295	18.8	0.89(0.77,1.03)^c^	0.024	64.5	
Genotyping method																	
PCR-RFLP	14	**0.81(0.69,0.96)** c	0.044	42.9	1.02(0.85,1.23)^c^	0.000	71.8	0.98(0.82,1.17)^c^	0.000	72.0	**0.87(0.80,0.94)**	0.444	0.3	0.94(0.87,1.02)^c^	0.009	53.4	
Taqman	3	**0.71(0.55,0.91)**	0.740	0.0	1.09(0.89,1.35)	0.099	56.7	0.97(0.80,1.18)	0.080	60.5	**0.69(0.57,0.85)**	0.903	0.0	**0.87(0.77,0.98)**	0.191	39.6	
PCR–LDR	3	1.14(0.91,1.44)	0.972	0.0	1.23(1.00,1.51)	0.287	19.8	1.19(0.98,1.45)	0.517	0.0	0.98(0.82,1.16)	0.576	0.0	1.05(0.94,1.18)	0.994	0.0	

CRC: colorectal cancer; HCC: hepatocellular carcinoma; PCR-RFLP: polymerase chain reaction–restriction fragment length polymorphism; PCR-LDR: ligation detection reaction.

a Number of included studies.

b *P* value of *Q* test for heterogeneity test.

c Random-effect model was used when P value for heterogeneity <0.05; otherwise, fixed-effect model was used.

Statistically significant results were in bold.

**Figure 4:**
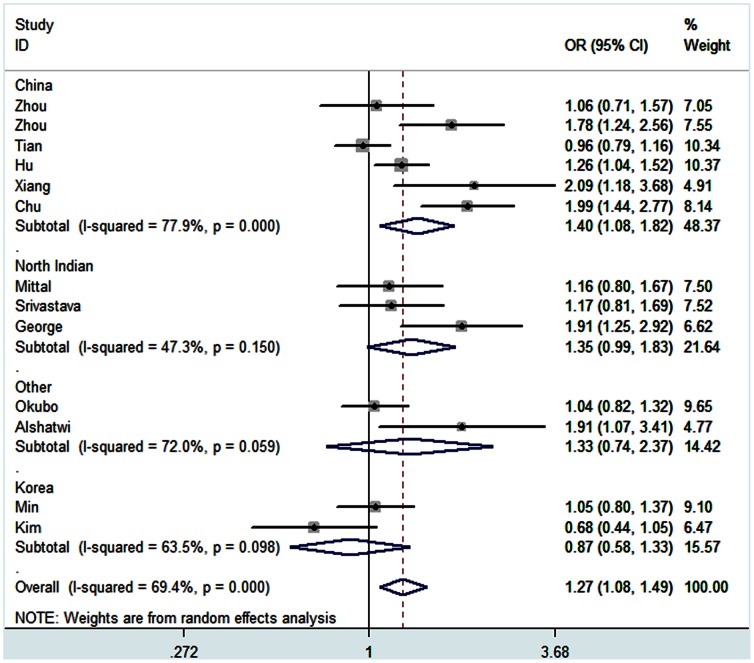
Forest plots of effect estimates for rs3746444 stratified by country (GG+GA vs AA). For each studies, the estimate of OR and its 95% CI is plotted with a *box* and a *horizontal line*. *Filled diamond* pooled OR and its 95% CI.

For rs3746444 polymorphism, an increased risk was revealed for the comparison of homozygote model (GG vs AA: OR = 1.25, 95% CI: 1.03–1.52, *P*_h_ = 0.073), heterozygote model (GA vs AA: OR = 1.28, 95% CI: 1.08–1.53, *P*_h_ = 0.000) and dominant model (GG+GA vs AA: OR = 1.27, 95% CI: 1.08–1.50, *P*_h_ = 0.000) (Figure 4) in the overall analysis. In the stratified analysis by cancer type, an increased risk was observed in breast cancer for the comparison of dominant model (GG+GA vs AA: OR = 1.31, 95% CI: 1.09–1.57, *P*_h_ = 0.182). Meanwhile, an increased risk was also found in other cancers (GA vs AA: OR = 1.32, 95% CI: 1.05–1.67, *P*_h_ = 0.000; GG+GA vs AA: OR = 1.29, 95% CI: 1.05–1.59, *P*_h_ = 0.001). In addition, sesults of subgroup analysis of country revealed increased cancer risk in China (GA vs AA: OR = 1.36, 95% CI: 1.06–1.75, *P*_h_ = 0.002; GG+GA vs AA: OR = 1.40, 95% CI: 1.08–1.82, *P*_h_ = 0.000; GG vs AA+GA: OR = 1.41, 95% CI: 1.06–1.87, *P*_h_ = 0.050) and North India (GG+GA vs AA: OR = 1.33, 95% CI: 1.07–1.66, *P*_h_ = 0.150). Similarly, an increased cancer risk association was observed in the subgroup analysis of source of controls. Subgroup analysis of population-based controls group showed the increased cancer risk for the comparison of heterozygote model (GA vs AA: OR = 1.27, 95% CI: 1.05–1.54, *P*_h_ = 0.000) and dominant model (GG+GA vs AA: OR = 1.24, 95% CI: 1.04–1.47, *P*_h_ = 0.002). Furthermore, subgroup analysis of hospital-based controls group showed the increased cancer risk for the comparison of homozygote model (GG vs AA: OR = 1.70, 95% CI: 1.09–2.67, *P*_h_ = 0.121) and recessive model (GG vs AA+GA: OR = 1.67, 95% CI: 1.07–2.61, *P*_h_ = 0.176), as summarized in Table 4.

**Table 4:** Stratified analyses of the miR-499A>G (rs3746444) polymorphism and cancer risk.

Variables	n^a^	GG vs AA			GA vs AA			GG+GA vs AA			GG vs AA+GA			G allele vs A allele			
		OR(95%CI)	*P* b	*I^2^*	OR(95%CI)	*P* b	*I^2^*	OR(95%CI)	*P* b	*I^2^*	OR(95%CI)	*P* b	*I^2^*	OR(95%CI)	*P* b	*I^2^*	
Total	13	**1.25(1.03,1.52)**	0.073	39.1	**1.28(1.08,1.53)** c	0.000	71.1	**1.27(1.08.1.50)** c	0.000	69.4	1.07(0.80,1.44)^c^	0.013	52.8	**1.18(1.04,1.34)** c	0.000	65.7	
Cancer type																	
HCC	3	1.25(0.36,4.34)^c^	0.023	73.6	1.00(0.76,1.31)	0.074	61.6	1.12(0.63,1.99)^c^	0.009	78.8	1.51(0.87,2.62)	0.062	64.0	1.13(0.64,2.01)^c^	0.001	85.0	
Breast cancer	2	1.50(0.98,2.30)	0.157	50.0	1.57(0.83,2.95)^c^	0.044	75.4	**1.31(1.09,1.57)**	0.182	43.8	0.97(0.29,3.19)^c^	0.019	81.7	**1.26(1.08,1.47)**	0.760	0.0	
Other cancers	8	1.14(0.90,1.44)	0.334	12.3	**1.32(1.05,1.67)** c	0.000	76.7	**1.29(1.05,1.59)** c	0.001	73.0	1.02(0.81,1.28)	0.092	43.0	**1.18(1.02,1.37)** c	0.006	64.9	
Country																	
China	6	1.54(0.93,2.56)^c^	0.029	59.8	**1.36(1.06,1.75)** c	0.002	74.0	**1.40(1.08,1.82)** c	0.000	77.9	**1.41(1.06,1.87)** c	0.050	54.8	**1.36(1.07,1.72)** c	0.000	79.8	
Korea	2	0.81(0.41,1.59)	0.366	0.0	0.94(0.74,1.19)	0.126	57.2	0.93(0.74,1.17)	0.098	63.5	0.83(0.42,1.62)	0.461	0.0	0.93(0.76,1.14)	0.103	62.4	
North India	3	1.02(0.70,1.50)	0.439	0.0	1.46(0.95,2.22)^c^	0.038	69.4	**1.33(1.07,1.66)**	0.150	47.3	0.83(0.58,1.18)	0.110	54.6	1.13(0.96,1.33)	0.726	0.0	
Other countries	2	1.27(0.82,1.97)	0.293	9.5	1.44(0.62,3.35)^c^	0.010	85.0	1.14(0.91,1.41)	0.059	72.0	0.91(0.32,2.58)^c^	0.040	76.3	1.11(0.93,1.33)	0.728	0.0	
Source of controls																	
Population based	11	1.17(0.94,1.44)	0.115	35.4	**1.27(1.05,1.54)** c	0.000	68.5	**1.24(1.04,1.47)** c	0.002	64.8	0.98(0.71,1.34)^c^	0.027	50.6	**1.14(1.00,1.29)** c	0.012	56.1	
Hospital based	2	**1.70(1.09,2.67)**	0.121	58.3	1.35(0.70,2.58)^c^	0.002	89.5	1.43(0.76,2.69)^c^	0.002	89.9	**1.67(1.07,2.61)**	0.176	45.4	1.44(0.82,2.52)^c^	0.002	89.7	

HCC: hepatocellular carcinoma.

a Number of included studies.

b *P* value of *Q* test for heterogeneity test.

c Random-effect model was used when P value for heterogeneity <0.05; otherwise, fixed-effect model was used.

Statistically significant results were in bold.

### Overall Effects for Alleles

Allele comparisons were also conducted in this meta-analysis. For allele comparison of rs2910164 polymorphism, a decreased cancer risk was observed in C allele (OR = 0.92, 95% CI: 0.85–1.00, *Z* = 2.00, *P* = 0.046, *P*_h_ = 0.000) for pooled analysis. In the subgroup analysis of cancer type, a decreased risk was observed in HCC (OR = 0.88, 95% CI: 0.78–1.00, *Z* = 1.99, *P* = 0.046, *P*_h_ = 0.245) and cervical cancer (OR = 0.72, 95% CI: 0.62–0.84, *P*_h_ = 0.796). Country subgroup analysis revealed C allele was associated with decreased cancer risk in Chinese population (OR = 0.87, 95% CI: 0.79–0.95, *P*_h_ = 0.000). When stratified analysis by genotyping method, C allele was associated with obvious decreased cancer risk by Taqman (OR = 0.84, 95% CI: 0.76–0.92, *P*_h_ = 0.570).

There was no evidence that rs11614913 T allele associated with the risk of cancer. Meanwhile we conducted subgroup analysis of cancer type, country, source of controls and genotyping method. In the subgroup analysis of cancer type, a decreased risk was observed in CRC (OR = 0.84, 95% CI: 0.76–0.92, *P*_h_ = 0.281) and lung cancer (OR = 0.87, 95% CI: 0.80–0.95, *P*_h_ = 0.854). Country subgroup analysis revealed T allele was associated with decreased cancer risk in Korean population (OR = 0.87, 95% CI: 0.79–0.96, *P*_h_ = 0.608). In the subgroup analysis of genotyping method indicated a decreased cancer risk with T allele determined by Taqman (OR = 0.87, 95% CI: 0.77–0.98, *P*_h_ = 0.191).

For rs3746444 polymorphism, a significant increased cancer risk was found in the population with G allele (OR = 1.18, 95% CI: 1.04–1.34, *P*_h_ = 0.000). In addition, cancer type subgroup analysis G allele was associated with increased breast cancer (OR = 1.26, 95% CI: 1.08–1.47, *P*_h_ = 0.760) and other cancers (OR = 1.18, 95% CI: 1.02–1.37, *P*_h_ = 0.006) risk. In the subgroup analysis of country, obvious increased cancer risk was observed in Chinese population (OR = 1.36, 95% CI: 1.07–1.72, *P*_h_ = 0.000). Meanwhile, borderline increased cancer risk was observed in population- based controls with G allele (OR = 1.14, 95% CI: 1.00–1.29, *Z* = 1.98, *P* = 0.047, *P*_h_ = 0.012).

### Test of Heterogeneity

For overall studies, there were significant heterogeneity observed in rs2910164, rs11614913 and rs3746444 polymorphisms. The source of the heterogeneity was evaluated for dominant model comparison by subgroups (cancer, country, source of controls and genotyping method). For rs2910164 polymorphism, the test revealed country (*χ^2^* = 11.64, df = 4, *P* = 0.020) but not cancer type (*χ^2^* = 11.03, df = 5, *P* = 0.051), source of controls (*χ^2^* = 0.05, df = 1, *P* = 0.832) and method (*χ^2^* = 4.54, df = 2, *P* = 0.103) contributed to substantial heterogeneity. For rs11614913 polymorphism, cancer type (*χ^2^* = 36.27, df = 5, *P* = 0.000) and country (*χ^2^* = 16.54, df = 3, *P* = 0.001) but not source of controls (*χ^2^* = 0.36, df = 1, *P* = 0.550) and genotyping method (*χ^2^* = 7.59, df = 3, *P* = 0.055) were found to contribute to substantial heterogeneity. For rs3746444 polymorphism, the source of heterogeneity was not observed in all subgroups.

### Sensitivity Analysis

Sensitivity analysis was performed to assess the stability of the results and assess the source of the heterogeneity by sequential removal of individual eligible study. For rs2910164 polymorphism, studies by Okubo [14] and Tian [10] were the main origin of heterogeneity. The heterogeneity was decreased when these two studies removed (CC+GC vs GG: *P*_h_ = 0.067, *I* ^2^ = 34.9%). For rs11614913 polymorphism, sensitivity analysis indicated that studies by Chu [15], Dou [16], George [17] and Srivastava [24] were the main origin of heterogeneity. The heterogeneity was decreased when these four studies removed (TT+CT vs CC: *P*_h_ = 0.054, *I* ^2^ = 38.4%). For rs3746444 polymorphism, sensitivity analysis indicated that studies by Tian [10], Chu [15], Kim [25] and Okubo [14] were the main origin of heterogeneity. The heterogeneity was decreased when these four studies removed (GG+GA vs AA: *P*_h_ = 0.066, *I* ^2^ = 45.4%). In addition, no other single study was observed to impact the pooled OR by sensitivity analysis.

### Publication Bias

**Figure 5:**
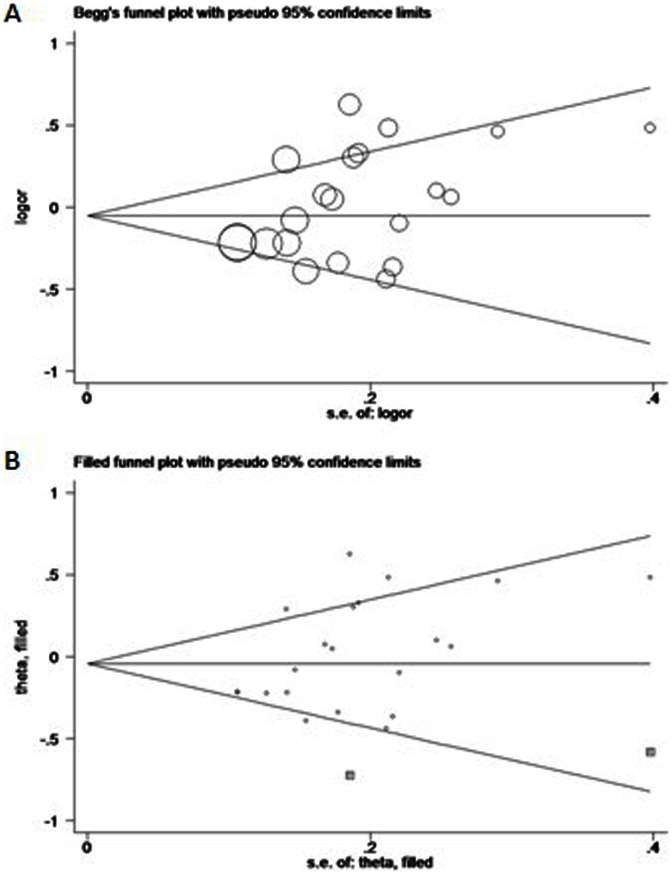
Begg’s funnel plot of Egger’s test for publication bias test for rs. Each *circle* represents as an independent study for the indicated association. Log[OR], natural logarithm of OR. *Horizontal lines* mean effect size. A: Begg’s funnel plot of publication bias test. B : Begg’s funnel plot of publication bias test after trim-and-fill method.

Begg’s funnel plot and Egger’s test were performed to assess the publication bias of enrolled literature. The shape of the funnel plot indicated obvious asymmetry in rs11614913 dominant model comparison (Figure 5A). Thus, Egger’s test was used to provide statistical evidence of funnel plot asymmetry (*t* = 2.15, *P* = 0.045) (shown in Table 5), which suggested the existence of publication bias in this meta-analysis. To adjust this bias, a trim-and-fill method illustrated by Duval and Tweedie [26] was utilized (Figure 5B). As a result, the conclusion with or without the trim-and-fill method did not change, which indicated that our results were statistically robust. While all models of rs2910164 and rs3746444 didn’t show any publication bias (*P*>0.05) (shown in Table 5).

**Table 5:** Egger’s test for three polymorphisms of miRNAs.

Polymorphism	Egger's test	Homozygote	Heterozygote	Dominant	Recessive
rs11614913	*t*	0.44	1.81	2.15	−0.92
	*P*	0.662	0.086	**0.045**	0.369
rs2910164	*t*	0.08	0.32	0.39	−0.32
	*P*	0.939	0.755	0.700	0.753
rs3746444	*t*	−0.36	1.86	1.6	−0.55
	*P*	0.727	0.089	0.138	0.590

Statistically significant results which means the shape of the funnel plot indicated obvious asymmetry were in bold.

## Discussion

As we all know, the association between the SNPs in protein-coding genes and the risk of cancer has been explained thoroughly, little cancer association studies concerning miRNA SNPs have been reported. In the present case-control study, associations of three miRNA polymorphisms (miR-146aG>C, rs2910164;miR-196a2C>T, rs11614913; miR-499A>G, rs3746444;) and cancer susceptibility were estimated. The polymorphisms of these three miRNAs may influence the effect of their targets, which contributed to the tumorigenesis, development and prognosis of many cancers. It is believed that tumor necrosis factor receptor-associated factor 6 and interleukin-1 receptor-associated kinase 1 are two potential targets of miR-146a [27], which could decrease the levels of these two proteins and reduce the activity of the NF-κB signaling pathway involving in tumorigenesis [28]. The main targets of miR-196a2 are homeobox (HOX) gene cluster and Annexin A1 (ANXA1). HOX genes include HOXB8, HOXC8, HOXD8, and HOXA7, which are known regulators of oncogenesis [29]. Meanwhile, ANXA1 is known as a mediator of apoptosis and an inhibitor of cell proliferation [30]. MiR-499 mainly targets to transcriptional repressor SOX6 [31], which reduce the level of fibroblast growth factor (FGF)-3 affecting cell proliferation and differentiation [32], [33]. In this meta-analysis, 31 eligible studies were enrolled to assess the association between three miRNA polymorphisms and cancer risk. We demonstrated that rs2910164 C allele, rs3746444 A allele and rs11614913 TT genotype were associated with significantly decreased risk of cancer.

Former studies regarding G to C variation in the miR-146a precursor showed that G-allelic miR-146a precursor displayed increased production of mature miR-146a compared with C-allelic and G allele in rs2910164 was associated with the predisposition of several cancers [18], [34], [35]. In overall pooled results from 21 studies, we concluded rs2910164 C allele was associated with decreased cancer risk. Furthermore, stratified analyses by cancer type revealed that rs2910164 CC genotype reduced risk of HCC, cervical cancer and prostate cancer, however no significant associations were observed in breast cancer and gastric cancer, which indicated that rs2910164 polymorphism might have different effects in distinct cancers. The results were consistent with the previous studies [13], [18], [36], [37]. While disaccords appeared in gastric cancer [14], [38], [39]. Inconsistent results might be caused by limited studies enrolled in this meta-analysis. Different study design and approach to select participants should also be taken into account. Followed stratified analyses by country indicated decreased cancer risk were discovered only in Chinese population, which reflected differences in genetic background and the environment exposured might produce different effects on cancer risk. At last, source of controls and genotyping method stratified analyses were also conducted in this meta-analysis. The results showed that different sources and methods could play different roles in cancer risk. As age and gender are risk factors for many cancers, which must be considered in this meta-analysis. The study conducted by Zeng et al [38] concluded that rs2910164 GG+GC genotype among males and subjects aged≤58 years was associtated with increased gastric cancer risk. The similar phenomenon was observed in the study by Zhou et al [39], which showed elevated gastric cancer risk was more evident among younger subjects (<65 years) with rs2910164 GG genotype. However, they observed no significant difference in the stratification of sex. As age increases, accumulated exposure to environmental carcinogens and genomic alterations would facilitate carcinogenesis [39]. Therefore, the age is believed to be an important risk factor for cancers, which was inconsistent with our results. More genomic alterations and environmental carcinogens may contribute to late-onset gastric cancer and stratification analysis by age should be more cautious. As for the pre-miR-146a sex-specific effect, the exact mechanism remains unclear. Therefore, well-designed, unbiased, large case-control studies were urgently needed to achieve a more accurately result.

Recently, rs11614913 polymorphism in pre-miRNAs has been reported to contribute to susceptibility of breast cancer [13], lung cancer [10], glioma [16], and influence survival of non-small cell lung cancer [40] by altering the expression of mature miR-196a and its binding to target mRNA. Our results showed that TT genotype was associated with decreased risk of CRC, breast cancer and lung cancer, which was consistent with previous findings [20], [23], [41], [42]. The controversy between our study were also apparent, no correlations were achieved between rs11614913 polymorphism and susceptibility of HCC and gastric cancer, while Li et al [12] and Okubo et al [14] presented contrary opinions respectively. In addition, contrast to Chinese and Korean population, rs11614913 CT genotype trend to increase cancer risk in North Indian population. Based on the above points, we deduced that cancer type and country differences made rs11614913 polymorphism have distinct effects. Cancer was a complex disease, and numerous factors would lead to tumorigenesis. Meanwhile, different cancers had different pathogenesis. Therefore, rs11614913 polymorphism might have distinct effects according to cancer types. Inconsistent results about different countries might be caused by differences in living habit, genetic background and the environment. In addition, this meta-analysis enrolled only 21 studies for rs11614913 polymorphism, inadequate study would be an influence factor. At the same time, the results also showed that different sources and methods contributed to different cancer risk. To further reveal the association between rs11614913 polymorphism and cancer risk, more well-designed studies based on homogeneous cancer patients and unbiased larger sample sizes were wanted.

As for rs3746444 G allele, an increased cancer risk was discovered in the pooled analysis. And subgroup analyses of cancer type and country showed that rs3746444 G allele association with increased risks were observed in breast cancer and Chinese population respectively. Meanwhile, a significant association was also observed for comparison of GG+GA vs AA in North Indian population. The results suggested different cancer types and countries could lead to distinct effects of rs3746444 polymorphism. Hu et al [13] showed that significantly increased breast cancer risk was associated with variant genotypes of hsa-mir-499 rs3746444 in Chinese women, which consistent with our results. Finally,source of controls stratified analysis was also conducted. The results indicated distinct source of controls was also an important influence factor to affect the association between rs3746444 polymorphism and cancer risk. However, the results were based on 13 studies enrolled in the analysis, which could affect the results owing to small amount of studies. To draw a more precise conclusion, more related studies needed.

According to the test of heterogeneity conducted above, it’s not difficult to perceive different countries contributed to heterogeneity of rs11614913 and rs2910164 polymorphisms, which indicated miRNAs might play different roles according to countries. Meanwhile, different cancer types were also a main factor contributed to heterogeneity. Significantly decreased associations were found mainly in HCC, lung cancer, cervical cancer, which suggested miRNAs might have different affections in different cancer types.

After all, this meta-analysis still existed some limitations. Firstly, publication bias which we have detected in rs11614913 polymorphism, and in other polymorphisms publication bias might also exist while we didn’t detect owing to quantitative restrictions of studies. Secondly, there was no uniform definition of controls, although most of the controls were mainly selected from healthy populations, a few of them were patients. Thirdly, the detailed information (such as age, sex, menstrual history, life-style and environmental factors) was not considered so that our unadjusted estimates should be confirmed by further studies.

In conclusion, this meta-analysis measured the association of three miRNA polymorphisms and cancer risk. We observed TT genotype of rs11614913 polymorphism was associated with decreased cancer risk, especially for CRC and lung cancer in Korean and North Indian population. Moreover, rs2910164 C allele was associated with decreased overall cancer risk especially for HCC, cervical cancer and prostate cancer risk in Chinese population. Whereas, rs3746444 G allele was a risk factor in Chinese population, especially for breast cancer. However, further studies based on larger, stratified population to facilitate evaluation the association between miRNAs and cancer risk.
